# Gaps in Prehospital Care for Patients Exposed to a Chemical Attack – A Systematic Review

**DOI:** 10.1017/S1049023X22000401

**Published:** 2022-04

**Authors:** Stephane Bourassa, Emmanuelle Paquette-Raynard, Daniel Noebert, Marc Dauphin, Pelumi Samuel Akinola, Jason Marseilles, Philippe Jouvet, Jacinthe Leclerc

**Affiliations:** 1. Sainte-Justine University Hospital Research Center, Montreal University, Montreal, Quebec, Canada; 2. Faculty of Medicine, Montreal University, Montreal, Quebec, Canada; 3. Retired - Canadian Armed Forces Intelligence Service; 4. Medical Intelligence CBRNE Inc., Quebec City, Quebec, Canada; 5. Library Services, Laval University, Quebec City, Quebec, Canada; 6. Retired - Royal Canadian Medical Service; 7. Department of Nursing, University of Quebec at Trois-Rivières, Trois-Rivières, Quebec, Canada; 8. Department of Nursing, Faculty of Health Sciences, University of Pecs, Pecs, Hungary; 9. Research Center, Quebec Heart and Lung Institute – Laval University, Quebec City, Quebec, Canada

**Keywords:** acute settings, chemical attack, chemical, biological, radiological, nuclear, explosive (CBRNE), decontamination, prehospital settings, protection, respiratory insults, treatment

## Abstract

**Introduction:**

The survivability of mass casualties exposed to a chemical attack is dependent on clinical knowledge, evidence-based practice, as well as protection and decontamination capabilities. The aim of this systematic review was to identify the knowledge gaps that relate to an efficient extraction and care of mass casualties caused by exposure to chemicals.

**Methods:**

This systematic review was conducted from November 2018 through September 2020 in compliance with Cochrane guidelines. Five databases were used (MEDLINE, Web of Science Core Collection, Embase, Cochrane, and CINAHL) to retrieve studies describing interventions performed to treat victims of chemical attacks (protection, decontamination, and treatment). The outcomes were patient’s health condition leading to his/her stabilization (primary) and death (secondary) due to interventions applied (medical, protection, and decontamination).

**Results:**

Of the 2,301 papers found through the search strategy, only four publications met the eligibility criteria. According to these studies, the confirmed chemical poisoning cases in acute settings resulting from the attacks in Matsumoto (1994), Tokyo (1995), and Damascus (2014) accounted for 1,333 casualties including 11 deaths. No study reported comprehensive prehospital clinical data in acute settings. No mention was made of the integration of specialized capabilities in medical interventions such as personal protective equipment (PPE) and decontamination to prevent a secondary exposure. Unfortunately, it was not possible to perform the planned meta-analysis.

**Conclusions:**

This study demonstrated gaps in clinical knowledge application regarding the medical extraction of casualties exposed during a chemical attack. Further research is required to optimize clinical practice integrating mixed capabilities (protection and decontamination) for the patient and medical staff.

## Introduction

Since the Aum Shinrikyo sarin nerve agent attacks in 1994 and 1995, respectively, civilian populations have been the target of chemical attacks.^1–7^ In their study, Ruckart, et al listed approximately 50 industrial chemicals that have the potential to be used in a terrorist plot against civilian populations.^8^ This, in conjunction with the existing threat posed by chemical warfare agents reported to act within seconds to hours,^9–15^ stresses the requirement to develop a medical preparedness capability.^16–21^ In the literature, there is a lack of medical guidelines and protocols for prehospital management in conjunction with the integrated use of protection and decontamination capabilities for both the health care professionals and the patients in the event of a chemical attack or other types of exposure (eg, biological, radiological, and nuclear).^10,22–30^ Furthermore, little is still known regarding the clinical impact of chemical exposures in humans. These knowledge gaps expose any population to inappropriate clinical care in the eventuality of a chemical attack with (or without) mass casualties, and therefore, a higher risk of death or long-term disability.^31,32^

The aim of this systematic review was to investigate the clinical knowledge and evidence-based practices applied in patients exposed to chemical weapons and treated in a prehospital or acute setting in order to identify the knowledge gaps that related to an efficient mass-casualty management in a contaminated environment. Ultimately, the objective was to compare the clinical outcomes of patients exposed to a chemical attack who received known interventions to reduce the risk of further contamination and progression of the harmful effects of the chemical (ie, protection, decontamination, and treatment).

## Methods

### Study Design

This study is a systematic review of the literature. The recommendations of the *Cochrane Handbook for Systematic Reviews of Interventions* were followed.^33^ The protocol was registered in the international register for systematic reviews maintained by the National Institute for Health Research (United Kingdom; PROSPERO Registration Number: CRD42019104473, Accepted on February 25, 2019; https://www.crd.york.ac.uk/prospero/; Last Update November 24, 2020).

### Source of Data

Online databases used for this study were: MEDLINE (US National Library of Medicine, National Institutes of Health; Bethesda, Maryland USA); Web of Science Core Collection (Thomson Reuters; New York, New York USA); Embase (Elsevier; Amsterdam, Netherlands); Cochrane (The Cochrane Collaboration; London, United Kingdom); and CINAHL (EBSCO Information Services; Ipswich, Massachusetts USA) from their inception through November 6, 2018. An update was performed on September 16, 2020 (Supplementary Material, Table S1; available online only).

### Search Strategy

Indexed and free-text terms, such as Respiratory, Warfare, and Chemical Threat, were selected by individually combining each of the two warfare modes with respiratory distress (Supplementary Material, Table S1). Afterwards, references were imported into the Covidence systematic review software (Veritas Health Innovation; Melbourne, Australia). Duplicate papers were automatically rejected by this software. Pre-trained individuals performed an abstract triage trial run on 40 selected references. Titles and abstracts were then independently screened by two reviewers and were retained for a full-text review if they met the inclusion/exclusion criteria listed in the next paragraph. Full texts of selected abstracts were then retrieved and assessed by two reviewers to confirm eligibility. At any point in the above-mentioned process, disagreements between reviewers were resolved using a consensus approach.

### Inclusion and Exclusion Criteria

**Figure 1.**
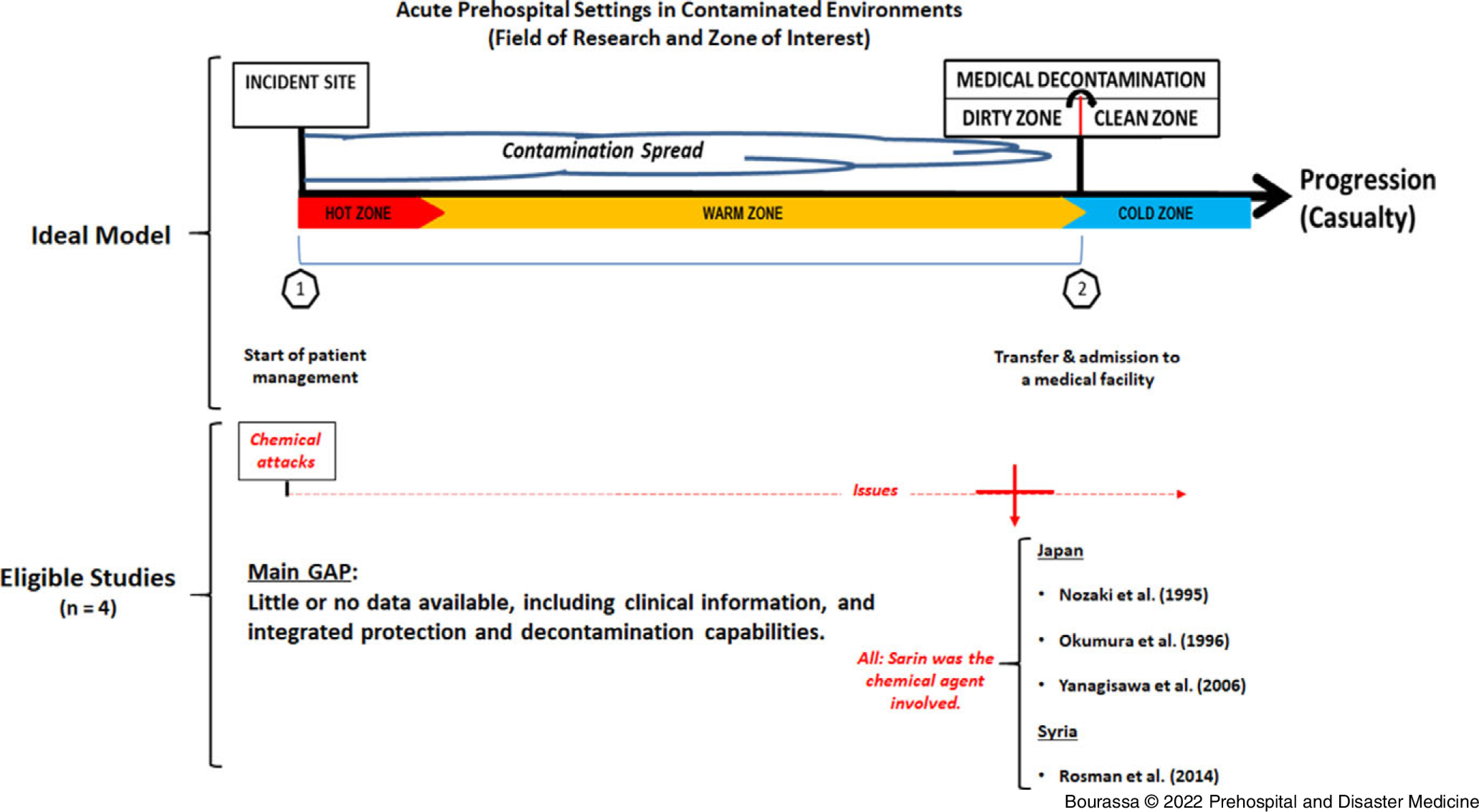
Illustration of the Field of Clinical Practice in Acute or Prehospital Settings in Contaminated Environments. Note: This is a summary of the zone of interest of this study (ie, from the incident site to the transfer of the patient in a clean zone, after being transported through the contamination environment, and then fully decontaminated). During a medical extraction from the contaminated environment (ie, hot and warm zones), the ideal mitigation measure against contaminants is facing upwind. Ideally, a very light decontamination process, called immediate decontamination, will be performed immediately after an attack/exposure to slow the agent’s absorption into the body. Thorough decontamination is a specialized process that occurs later, ideally prior to admission to a medical facility. Number 1 – Clinical process occurring from the moment the patient is handled until decontamination is completed; Number 2 – Continuity of care happening at the patient’s transfer, admission, and beyond within a medical facility (eg, emergency room or intensive care unit).

Inclusion criteria were: (1) exposure to a chemical incident (eg, mass casualties); (2) chemical known to affect the respiratory system; (3) interventions involving the assessment of a triad of integrated key competences (protection for staff and patients, decontamination, and treatments); (4) patient outcomes (ie, primary: patient’s health condition remaining stable due to medical, protection, and decontamination interventions; secondary: patient’s mortality occurring at his/her admission despite medical, protection, and decontamination interventions); (5) studies with original data, including those conducted on animals induced with chemical agents in order to simulate a medical extraction of casualties; and (6) studies should have occurred within the zone of interest. The zone of interest where medical interventions took place in eligible studies was defined as the casualty extraction from the incident site where the chemical attack occurred to the clean zone where the patient was admitted to the hospital (Figure 1).

Studies were excluded if: (1) effects were shown on insects, plants, or materials; (2) procedures were performed in a clean/cold zone setting once the patient was fully admitted and handled by the medical facility’s staff; (3) they did not address a respiratory disorder; (4) they did not present original data (eg, reviews); or (5) the topic was not related to a chemical threat (eg, suicide attempt).

### Quality Appraisal/Risk of Bias

Two quality appraisal charts were used in order to detect and mitigate the variability in staffers’ assessments. The first was developed by Hong, et al from McGill University (Montreal, Quebec, Canada;^34^ the second was from Hawker, et al (Appendices C and D from that research).^35^ The risk of bias in each eligible study was assessed independently by two reviewers.

### Extraction of Data

Data extraction was performed independently by two individuals. Extracted data were imported into an Excel (Microsoft Corp.; Redmond, Washington USA) spreadsheet format developed in-house based on Cochrane and Covidence models (Supplementary Material, Table S2; available online only).

### Synthesis of Evidence

The method for qualitative synthesis of evidence that was used led to produce different summaries: (1) health management plan and clinical tools used to respond to a chemical attack; (2) detection of toxidromes in the patient’s condition versus the clinical intervention provided; (3) delays to response; and (4) association between these variables. Further details are found in the Supplementary Material (Body Text; available online only).

### Biostatistical Analysis

Descriptive statistics were planned to summarize study characteristics, including mean and standard deviations (SD) and median and interquartile range (IQR) and proportions, according to the type of data. A Student’s t-test was planned to compare the clinical onset of chemical agents and algorithms of treatment, along with forest plots to highlight the difference between each agent’s action mechanism and therapy onsets. To mitigate the potential impact of missing data, an imputation model was planned (root mean square error). Descriptive statistics and other numbers were to be computed with IBM SPSS Statistics Software (SPSS Inc.; Chicago, Illinois USA) and StatsDirect statistical software (StatsDirect Ltd.; Sale, Cheshire, United Kingdom). A meta-analysis involving the use of a random effects linear model (mixed effects model) was planned to correlate the effect of a studied chemical agent with one of the clinical interventions made by health care professionals. This would have highlighted the windows of treatment opportunities in such contaminated environments (RevMan software version 5.3; The Cochrane Collaboration Network; London, United Kingdom). The statistical significance level was set at P <.05 and interpreted with 95% confidence intervals (CI). These biostatistics plans had been reviewed by a biostatistician. Unfortunately, it was not possible to run any statistical analysis due to the heterogeneity of eligible studies and paucity of extractable data.

## Results

**Figure 2.**
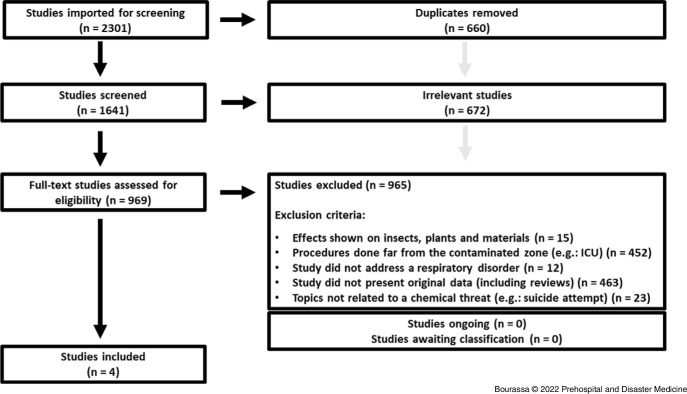
PRISMA Diagram. Abbreviations: PRISMA, Preferred Reporting Items for Systematic Reviews and Meta-Analyses; ICU, intensive care unit.

The flowchart Preferred Reporting Items for Systematic Reviews and Meta-Analyses (PRISMA) diagram is presented in Figure 2. A PRISMA checklist was also used based on the *Prehospital and Disaster Medicine* journal’s instruction for authors^36^ (Supplementary Material, Table S3; available online only). After title and abstract screening, 969 of the 1,641 studies identified through the search strategy remained eligible for full-text assessment. In the end, only four studies (all related to a sarin gas attack) were eligible for inclusion in this systematic review.^37–40^ No further studies were added after the update performed in September 2020.

Eligible studies^37–40^ reported retrospective data on patients or health care professionals treated in a contaminated cold zone (eg, medical facilities) and contained some information related to the acute settings (Figure 1). The patients included in these four studies were victims of three different events: (1) the 1994 Matsumoto suburban terrorist attack (Japan);^40^ (2) the 1995 Tokyo subway terrorist attack (Japan);^37,38,40^ and (3) the 2013 Damascus civil war attack (Syria).^39^ In the case of the Tokyo attack, one paper reported some data^40^ that were present in two others.^37,38^ In this analysis, information was considered common to two or more of the studies as a single data set. In other words, matching results were treated as a single response, but when different results were presented, these accounted for two independent medical responses and are reported as such in this paper.

### Quality Appraisals

The quality appraisals are presented in Table S4 and Table S5 (Supplementary Material; available online only) of the supplement. Overall, eligible studies showed a moderate to high risk of bias. The two tools used provided similar results.

### Subjects Characteristics and Outcomes

A summary of each study is presented in Table 1. Based on the limited available data scattered throughout the eligible studies,^37–40^ this study estimates that a minimum of 8,550 individuals were exposed during the sarin gas attacks that struck Japan and Syria, of which 1,333 casualties and 11 deaths were confirmed medical cases managed on the day the attacks occurred (Supplementary Material, Table S6 and Table S7; available online only). The 1,333 casualties represented confirmed chemical poisoning cases in acute settings and were considered in this study as the number of included patients.

**Table 1.** Summary of Included Studies

Study	Attack (s)	n (acute cases)	Method	Main Results & Outcome(s)	Protection	First-Aid	Means of Decontamination & Existence of Specialized Assets (Ambulatory and Medical: Yes/No)	Clinical Treatments (ie, until transfer)
Nozaki, et al (1995)^37^	1995 Tokyo Subway (Terrorism: Aum Shinrikyo; CWA: Sarin) *	15	Design & Source of data: Retrospective-Observational study in which medical records were used. ^£^ Measurement(s): Assessed risks of secondary exposure in health care staff. ^†^ ^‡^	Main Result(s): Secondary exposures caused mainly by contaminated patients (ie, vector). Main outcome(s): Recommendation for prompt decontamination and treatments.	Not reported ^€^	Not reported	Means used: Partially reported. Specialized assets (Ambulatory and Medical): No. ^§^	Partially reported
Okumura, et al (1996)^38^	1995 Tokyo Subway (Terrorism: Aum Shinrikyo; CWA: Sarin) *	640	Design & Source of data: Retrospective-Observational study in which medical records were used. ^∫^ ^£^ Measurement(s): Provided a description of graded intoxication cases; no other detail explicitly provided by the authors. ^†^ ^‡^	Main Result(s): 111 of 640 cases were characterized as moderate to severe. Main outcome(s): Mass casualty response capability for future disaster plans along with improvements to on-call resources were suggested.	Not reported	Partially reported, except for one CPR case.	Means used: Partially reported. Specialized assets (Ambulatory and Medical): No. ^§^	Partially reported
Rosman, et al (2014)^39^	2014 Syrian population attacked in Damascus (Civil war: Air strike by Assad; CWA: Sarin). *	130	Design & Source of data: Retrospective-Observational study based on YouTube footage revealing clinical information about patients. ^∫^ Measurement(s): Assessed the clinical response for intoxicated cases observed in 67 analyzed videos. ^†^ ^‡^	Main Result(s): 91.5% of cases were defined as moderate or worse; most suffered from dyspnea. A severe lack of antidotes and medical resources was observed. Main outcome(s): social media footage may improve future preparedness and readiness of health systems for various disasters; particularly in dealing with mass casualty events.	Partially reported ^§^	Not reported	Means used: Partially reported. Specialized assets (Ambulatory and Medical): No. ^§^	Partially reported
Yanagisawa, et al (2006)^40^	1994 Suburban Matsumoto & 1995 Tokyo Subway (Terrorism: Aum Shinrikyo; CWA: Sarin). *	>1203^!^	Design & Source of data: It was a mixed study design (Retrospective-Observational for acute effects & Longitudinal-observational for the post-attack health effects). Their data sources were patient interviews and medical records. ^∫^ Measurement(s): Health effects (physical and mental) (duration of up to 10 years after the acute phase). ^†^ ^‡^	Main Result(s): Other long-term effects (physical & psychological) lack of data in acute settings were reported. Main outcome(s): Teams of neurologists equipped with neurotoxic diagnostics and intervention protocols must be developed and included in preparedness plans.	No-information confirmed (M; T) ^§^	Not reported (M; T)	Means used: Not-reported (M; T). Specialized assets (Ambulatory and Medical): No. ^§^	Partially reported (M; T)

Abbreviations: CPR, cardiopulmonary resuscitation; CWA, chemical warfare agents; ED, emergency departments; M, Matsumoto; T, Tokyo.

* Unawareness of a CWA attack.

∫ – Design deduced from the paper as authors did not specify their design.

£ – The source of data is deduced from the paper as it was not provided by the authors.

† – Measurement not substantiated in the literature.

‡ – No biostatistics plan and analysis.

! – This represents the minimum number of patients managed by medical authorities over the years above the numbers treated in acute settings and reported in this paper.

€ – Secondary exposures confirmed by authors (ie, expansion of the contamination zone due to contaminated carriers [casualty/vehicle]).

§ – Signs of secondary exposures (ie, issues with PPE and decontamination capabilities, health care staff, and other rescuers becoming sick or absence of specialized capabilities).

¥ – Visual Analogue Scale Grade (No information confirmed – Absence of information about the topic/category confirmed; Not reported – Uncertainty as to whether the authors might or might not have analyzed this topic/category; Partially – little information available; Detail(s) provided – Disclosure of the information).

### Overview of the Populations Treated in Acute Settings

Nozaki, et al was the only study to provide a complete basic breakdown of the affected population (n = 15 medical staff members; 13 males, two females, all Japanese, ages ranging from 25 to 51 years old).^37^ In the Okumura, et al study, 640 patients were treated but the authors only provided a partial breakdown (395 males, five pregnant females; aged eight to 65 years old).^38^ No information was provided on the remaining 240 individuals poisoned.^38^ In their study, Yanagisawa, et al reported that the 1994 Matsumoto attack resulted in a total of seven fatalities and 272 casualties treated the day of the attack (264 patients; eight rescuers).^40^ In the 1995 Tokyo attack, the same authors reported four dead and 920 survivors treated the day of the attack: (1) St. Luke’s Hospital: 750 (one dead; 749 affected individuals, 639 patients and 110 medical staff members); (2) Keio University: one dead, 85 patients, 15 medical members; (3) Teishin Hospital: 32 patients and 39 rescuers; and (4) Tokyo Subway Station: two dead.^40^ Age and gender were not reported.^40^ Rosman, et al provided a casualty estimate (n = 130; 3% females, 97% males; of which 60% were children) based on their source of data (YouTube [Google, Inc.; San Bruno, California USA] social media footage analysis).^39^

### Medical Interventions During Casualty Extraction

None of the four papers^37–40^ provided comprehensive details regarding treatments given to patients as a function of symptomatology during the medical extraction. In the Nozaki, et al study, no details were provided regarding the medical interventions performed on the 85 contaminated patients upon arrival at Keio University Hospital. In addition, their clinical presentation during the medical extraction from the chemical attack site in Tokyo and once admitted to hospital was not reported by the authors.^37^ However, the authors did report some information on two patients’ respective health conditions, one convulsive and one in cardiac arrest, for which cardiopulmonary resuscitation (CPR) was performed upon the transfer to the emergency department (ED)/emergency room (ER).^37^ Table 2 lists the available information related to the continuity of care provided by the Japanese medical centers at the patient’s admission.

**Table 2.** Listed Treatments Patients Received Once Admitted

Study	Chemical Incident	Treatments	Remarks
Yanagisawa, et al (2006)^40^	Matsumoto Incident (1994)	1. Atropine sulfate; 2. Benzodiazepines; 3. Intravenous fluids; 4. Ventilation; 5. Intubation.	Atropine was given in large quantities to treat sarin-induced miosis.
	Tokyo Area Incident (1995; St-Luke’s)	1. Pralidoxime iodide (PAM); 2. Intravenous diazepam; 3. Mechanical ventilation.	
	Tokyo Area Incident (1995; Keio University Hospital)	Atropine sulphate or oximes.	Elsewhere in the paper, authors also indicated that PAM and 2-PAM were administered intravenously from two to six hours after the sarin exposure without specifying to which medical centers they were referring.
Okumara, et al (1996)^38^	Tokyo Area Incident (1995; St-Luke’s)	1. Atropine up to 9 milligrams (mg); 2. Either 2-PAM or up to 800mg of a pralidoxime; 3. Diazepam up to 30mg; 4. Tropicamide; 5. Phenylephrine hypochlorite; 6. Steroidal eye drops; 7. Antidepressants; 8. Intubation; 9. Ventilation.	The entire casualty management effort at St. Luke’s the day of the attack, that are detailed differently than those found in Yanagisawa, et al (2006).

Note: There was no indication on the use of oxygen found in these studies.

Abbreviations: PAM, pralidoxime iodide; 2-PAM, 2-pyridinealdoxime methiodide.

In Okumara, et al, one person performed CPR on a victim at the site of the chemical attack in Tokyo before getting poisoned by sarin herself. At her arrival at St. Luke’s ER with two other victims, that Samaritan also was in cardiac arrest.^38^ Regarding medical extraction, the authors only reported the transportation performed by paramedics.^38^ No information was provided on the medical interventions performed on 99 patients during their transport to hospital by first responders.^38^ Similarly, the authors did not report on first aid performed by good Samaritans or health care staff for the remaining 541 rescued patients.^38^

Yanagisawa, et al reported that all cardiac arrest patients from Matsumoto (1994; n = 3) and Tokyo (1995; n = 5) were treated upon arrival to the ED, but no further detail was provided.^40^

In Rosman, et al, the authors listed treatments provided to patients in non-medical facilities: atropine, steroids, furosemide, supplemental oxygen (O_2_), nasopharyngeal suctioning, bag valve ventilation, tracheal intubation, mechanical ventilation, and chest compression.^39^ They noted that standard monitoring equipment was not used to measure O_2_ saturation, blood pressure, or cardiac electrical activity.^39^ As noted by the authors, all medication was administered intravenously with no evidence of autoinjector use.^39^ Moreover, they casted doubt on the authenticity of 66 out of 67 YouTube videos analyzed.

The two studies of the 1995 incidents that occurred in Japan^38,40^ did not report whether the delivery procedures or special gestational care successfully preserved the life of the fetus/newborn or not. Similarly in the Damascus incident, Rosman, et al only mentioned that children accounted for 60% of the 130 casualties.^39^ Okumara, et al reported an eight-year-old victim as the youngest casualty treated at St. Luke’s Hospital, but no further clinical information was provided.^38^ Likewise, no information was reported for specific populations.

### Medical Interventions Due to Secondary Exposure in Rescuers and Medical Staff

Nozaki, et al was the only study^37^ of the four^37–40^ to have reported a medical response involving medical staff affected by managing patients contaminated by sarin, which led to a secondary exposure or the relocation of the contaminated zone to an unprepared location. The authors reported a six-hour wait before medical staff received confirmation that sarin gas was the cause of patient intoxications.^37^ In the interim, medical staff provided medical care for an unknown exposure.^37^ The authors briefly described some close-contact events that occurred between 15 clinicians and 85 contaminated patients.^37^ Of these cases, only two were summarily described (the medical management of one convulsive and one cardiac arrest case). The authors also enumerated 14 of 15 reported total cases involving medical staff according to the following categories: four cardiac arrests, two intubations, three cases of contamination, four unspecified tasks, and one observation. Of these, six adult caregivers received atropine (0.5-1.0mg intramuscular); one caregiver received 2-PAM (500mg).^37^ Other causative factors that led to a secondary exposure are covered in the Results section and the Supplementary Material (Body Text; available online only).

### Medical Algorithms

Throughout the four studies,^37–40^ there was no indication that specific chemical intoxication algorithms or clinical guidelines for patient management were used, except for triage.^37,39^ Medical authorities in Matsumoto^40^ (1994) and medical staff at St. Luke’s Hospital (1995) used an algorithm^38^ for patients’ triage (mild, moderate, and severe) and management,^38,40^ even if the exact terms used varied. It should be noted that even though the two studies partly covered the same medical response at St. Luke’s Hospital,^38,40^ their respective authors did not report precisely the same version of the triage score. No gold-standard reference related to that triage score was found in either study.^38,40^ The definitions are shown in Table S8 of the Supplementary Material (available online only).

### Immediate and Specialized Decontamination Capabilities

None of the four papers^37–40^ reported whether or not immediate decontamination procedures were performed during the extraction process before their arrival at a specialized decontamination asset before admission to a medical facility, usually considered as a clean zone. None of the papers reported the existence of specialized assets capable of combining actions like continuing medical treatments, performing decontamination, and ensuring safety while wearing personal protective equipment (PPE).^37–40^

However, three papers^37–39^ out of the four^37–40^ provided details on some of the decontamination means used on patients while at the medical facilities. Nozaki, et al reported: (1) ventilation of resuscitation rooms was ensured by opened doors and windows; and (2) contaminated belongings were placed in sealed vinyl bags.^37^

Okumara, et al summarized decontamination steps as the removal of contaminated clothing.^38^ They also reported that patients were either showered or bathed depending on their state of consciousness, but provided no further detail.^38^ In the case of Rosman, et al, their observation of procedures was reported as: (1) wash out with water, which included rubbing the casualty’s face and chest (25% of videos); and (2) full removal of clothing (10 out of 67 videos in which decontamination took place at medical facilities with no additional information provided).^39^

### Personal Protective Equipment

None of the four studies confirmed PPE was worn during the management of the chemical attacks.^37–40^ Rather, while two studies made no mention of PPE for rescuers, the clinicians, and the patients,^37,38^ the two remaining studies presented little information on their means of protection.^39,40^ The authors reported that health care professionals were not protected from contamination despite the suspicion of gas poisoning.^40^ Regarding the Tokyo attack, the authors confirmed rescue staff did not use special PPE to protect themselves against the gas exposure.^40^ They neither specified the members of the rescue teams nor if the medical staff used any PPE.^40^ In Rosman, et al, it was reported that no PPE was worn other than the sporadic use of latex gloves and surgical masks by medical staff (10 out of 67 videos).^39^

### Other Causes of Secondary Exposures

As previously indicated, Nozaki, et al^37^ was the only study of the four^37–40^ that covered the medical management of new patients (ie, medical staff) due to secondary exposures induced by contaminated patients. For its part, Yanagisawa, et al identified failures in PPE capabilities as a cause of secondary exposure to rescuers for the Matsumoto and Tokyo chemical attacks.^40^

### Meta-Analysis

Due to the paucity of studies and the heterogeneity of the data, no meta-analysis was performed.

## Discussion

### Major Findings

In this systematic review, results showed that very few studies reporting on acute medical care after a chemical terrorist attack or civil war clash have been published so far.^37–40^ No clinical data were found regarding mass-casualty management from the incident site to the point of transfer at a medical facility (ie, acute settings). According to available information, the treatments delivered to victims were very heterogeneous and no dedicated algorithm was used. Also, there were major protection and decontamination capability deficiencies (eg, standardization, equipment, and their application in medical interventions) for both patients and staff. These led not only to secondary contamination of health care professionals and medical facility environments, but may also have played a role in the worsening of patients’ conditions.

One study identified by the search strategy concerned the 2014 chemical attack in Syria,^39^ while the remaining three^37,38,40^ addressed the 1994 and 1995 events in Matsumoto and Tokyo, respectively. Lack of detail regarding medical interventions reported by the authors^37–40^ hindered the ability to assess the adequacy of the interventions performed on patients as no mention was made of gold standards, guidelines, or protocols. In some instances, only resuscitation maneuvers were reported,^37,38,40^ and no information were provided regarding PPE and decontamination capabilities for patients, rescuers, or health care professionals.^37–40^

Due to the modest quantity and quality of the studies identified by the search strategy and the heterogeneity of the data, researchers were unable to proceed with the biostatistical analysis plan. This situation was not precedent-setting as in McGaughey, et al, a systematic review conducted on an early-warning system, experienced similar challenges with two included studies (ie, showing poor evidence, impossible to make comparisons).^41^

### Importance of Medical Algorithms, Treatment Capabilities, and Disaster Plan

With the exception of three studies which showed that similar triage systems were used in the management of casualties during the chemical attacks in Japan,^38–40^ the use of a medical algorithm or a clinical guideline was not reported in selected studies.^37–40^ It should also be noted that Okumura, et al reported triage categories using terms more directly related to the clinical response,^38^ while Yanagisawa, et al simply listed the definitions with barely any clinical detail regarding the events in Matsumoto and at St. Luke’s hospital in Tokyo.^40^

Only one study mentioned the activation of the disaster plan at St. Luke’s Hospital,^38^ which also strengthens the argument concerning a complete lack of preparedness to deal with such disasters. Most importantly, the means of treatment and the overall capability during the medical extraction of patients from the incident site to their transfer to the ER, presumably after a thorough decontamination, was not reported.^37–40^ The decontamination aspect is of particular importance in situations where secondary exposures occurred in rescuers and medical staff at unprepared locations.^37–40^ At first glance, this suggests that algorithms for clinical response in acute settings or during an extraction within a contaminated environment need to be further developed, more widely disseminated, and regularly updated. However, the passage of time between the attacks, the publication of the related studies, and present-day knowledge and recommendations available in the grey medical literature of several organizations render comparisons fruitless. This nonetheless also suggests that recommended medical practices should, on the one hand, be subjected to more scrutiny in order to integrate medical developments and innovations such as O_2_ therapy, and should also, on the other hand, focus on the application of novel technologies in the acute settings field of research, including capabilities offered by artificial intelligence. Thus, this could be envisioned as a research study in itself, or even an entire research program.

### Importance of Protection and Decontamination Capabilities

Throughout the four papers^37–40^ analyzed, no information was provided concerning the provision of a certain level of protection to the patient with adapted protective gear such as the casualty bag used by North Atlantic Treaty Organization (NATO; Brussels, Belgium) nations since the Cold War^42^ in order to prevent secondary caregivers’ exposure and to mitigate contaminant absorption due to residual contaminants on the patients’ clothes. Decontamination capability information was also lacking.^37–40^ These gaps suggest that medical algorithms, protective equipment, and decontamination processes in acute settings in the context of a mass-casualty event due to a chemical attack need to be implemented concurrently.

Most of the events studied occurred years ago (more than 25 years for Japan and seven for Syria). Despite this, and the numerous chemical attacks that took place during the Iran-Iraq war (1983-1988), a lack of publications, applied clinical knowledge, and evidence-based practices still exists when it comes to ensuring in-depth and efficient protection and decontamination for the patient and the clinician. In the literature, studies regarding protection have mostly focused on responders and medical staff PPE.^43–45^ Very little attention has been paid to patients.^3,9,30,46,47^ To the authors’ knowledge, few studies have investigated the integration of medical devices in PPE^3,12,47–49^ for quicker clinical responses.^3,12^ Regardless of the wearer, PPE does not allow for easy access to monitor vital signs or initiate medical interventions such as respiratory and hemodynamic management. It also seems that consideration has yet to be given to populations such as pregnant women, children, and patients with psychiatric, acute, and chronic illnesses. It should be noted that decontaminating a patient is expected to be a complex specialized task best performed by a trained clinician. This can, for example, entail combining decontamination techniques with the safe use of decontaminants and equipment, and most importantly, adjusting patient treatment as required in response to their deteriorating condition or specific injuries (eg, cardiac arrest or open wounds).

## Strengths and Limitations

This study’s strength is the exhaustivity of the literature analysis. However, the study also has limitations. Its results may have been subject to a publication bias due to inaccessible classified information that, unbeknownst to the authors, may still exist in Japan or within international organizations such as the World Health Organization (WHO; Geneva, Switzerland), the Organization for the Prohibition of Chemical Weapons (OPCW; The Hague, Netherlands), and the United Nations Office for Disarmament Affairs (UNODA; New York USA). Despite the doubts cast by Rosman, et al regarding the authenticity of the YouTube footage following a chemical attack in Syria,^39^ this current study was not able to confirm whether the results were prejudicially biased, which could have induced a selection and an information bias. The studies selected reported data on chemical attacks that occurred more than 10 to 20 years ago. Patient management has evolved, especially with the increased awareness of PPE since the COVID-19 pandemic. Nevertheless, the limited number of studies with a moderate risk of bias as well as the heterogeneity of their methods and results may have hindered the ability of this study to draw any firm conclusion.

## Conclusion

This systematic review demonstrates gaps in clinical knowledge and protection and decontamination capabilities concerning the medical extraction of casualties exposed to a chemical attack. Therefore, further research is required to optimize a clinical practice integrating mixed capabilities (protection and decontamination) for the benefit of patients and medical staff.

## Supplementary material

For supplementary material accompanying this paper visit https://doi.org/10.1017/S1049023X22000401. click here to view supplementary material
