# Supplementary material for: Gaps in Prehospital Care for Patients Exposed to a Chemical Attack – A Systematic Review
Source: Prehosp Disaster Med. 2022 Mar 11;37(2):230–9. doi: 10.1017/S1049023X22000401 (PMC8948487; doi:10.1017/S1049023X22000401)
Supplement: Supplementary file 1 [file S1049023X22000401sup.zip › S1049023X22000401sup005.docx]

Table S4 and Table S5 show quality appraisal results.

| **STUDY** | **SCREENING QUESTIONS** | | **QUANTITATIVE DESCRIPTIVE STUDIES** | | | | |
| --- | --- | --- | --- | --- | --- | --- | --- |
|  | Are there clear research questions? | Do the collected data allow to address the research questions? | Is the sampling strategy relevant to address the research question? | Is the sample representative of the target population? | Are the measurements appropriate? | Is the risk of non-response bias low? | Is the statistical analysis appropriate to answer the research question? |
| Nozaki et al. (1995) | No | Can’t tell | Can’t tell | No | No | Can’t tell | No |
| Okumura et al. (1996) | No | Can’t tell | Can’t tell | Can’t tell | Can’t tell | Can’t tell | Yes |
| Rosman et al. (2014) | No | Can’t tell | Can’t tell | No | No | Can’t tell | No |
| Yanagisawa et al. (2006) | No | Can’t tell | Yes | Yes | No | Can’t tell | No |

**Table S4. Hong’s mixed methods appraisal tool applied to published papers retained.**

**NOTE.** Hong et al. (2019) Appendix 1 template format was used. Answer choices were Yes, No, Can’t Tell. (Hong QN, Pluye P, Fàbregues S, Bartlett G, Boardman F, Cargo M, et al. *Improving the content validity of the mixed methods appraisal tool: a modified e-Delphi study*. Journal of Clinical Epidemiology 2019; 111:49-59.e41) ^4^

| **Study** | **Abstract & Title** | **Introduction & Aims** | **Method & Data** | **Sampling** | **Data analysis** | **Ethics & Bias** | **Findings & Results** | **Transferability & Generalizability** | **Implications & Usefulness** |
| --- | --- | --- | --- | --- | --- | --- | --- | --- | --- |
| Nozaki et al. (1995) | Fair | Poor | Poor | Poor | Very Poor | Very Poor | Poor | Poor | Good |
| Okumura et al. (1996) | Very Poor | Poor | Very Poor | Poor | Very Poor | Very Poor | Poor | Fair | Good |
| Rosman et al. (2014) | Fair | Fair | Poor | Good | Poor | Good | Poor | Poor | Good |
| Yanagisawa et al. (2006) | Poor | Poor | Very Poor | Fair | Poor | Very Poor | Poor | Poor | Good |

**Table S5. Hawkers’ appraisal tool applied to published papers retained.**

**NOTE**. Hawker et al. (2002) Appendix D template format was used. Answer choices were Good, Fair, Poor, Very Poor. (Hawker S, Payne S, Kerr C, Hardey M, Powell J. *Appraising the Evidence: Reviewing Disparate Data Systematically*. Qualitative Health Research 2002;12(9):1284-1299.) ^5^

Table S6 and Table S7 show results of the data we processed in order to obtain uniform data on counts for exposed and deceased individuals from the limited and scattered data available across the four studies ^6-9^.

|  | **Impact of the Chemical Exposure** | | |  | **Day of the attacks** |
| --- | --- | --- | --- | --- | --- |
| **Country** | **Affected** | **Dead** | **Total exposed (minimum)** | **Authors** | **Confirmed cases managed** |
| **Japan**  **(1994 & 1995) *** | > 6000 | 19 | 6019 | Yanagisawa et al. (2006) ^9^ | 1203 **^‡^** |
| **Syria**  **(2014) †** | > 1130 ***** | 1400 ***** | 2530 | Rosman et al. (2014) ^8^ | 130 **^€^** |
|  |  | **Grand Total** | 8549 (rounded to 8550) |  | 1333 |

**Table S6.** **Calculation of cases of exposure to sarin gas per country and grand total.**

**Acronyms and Symbols**:

***** Since Yanagisawa et al. (2006), presented more accurate statistics on the chemical events, they superseded those from Okumura et al. (1996). Okumura et al. (1996) reported different statistics for the Tokyo attack (i.e.: 11 dead, more than 5000 exposed individuals required medical attention) ^7^. Yanagisawa et al. (2006), for their part, reported 7 died in Matsumoto in 1994, while 12 died in Tokyo in 1995 (2 at the attack site (Station Yard), 10 in hospitals - an hour to three months after the attack) ^9^.

**^†^** In Rosman et al. (2014), the authors analyzed 130 confirmed cases the day of the attacks using YouTube footage ^8^ . The authors reported with uncertainty that affected people received care in medical and non-medical facilities ^8^. They also presented data from third-party organizations, such as the United Nations (1400 dead and over 1000 exposed) ^8^.

**^‡^** Same data as Table S7 for the day of the 1994 and 1995 attacks in Japan. Yanagisawa et al. (2006) presented numbers from the Japanese medical centers involved in the response to the chemical attacks, such as in Matsumoto and Tokyo ^9^, as opposed to two studies that only addressed cases seen at Keio University and St. Luke’s Hospital medical facilities in the Tokyo area (Novaki et al. (1995); Okumura et al. (1996)) ^6,7^.

**^€^** Same data as Table S7 for the day of the 2014 attacks in Syria addressed by Rosman et al. (2014) ^8^.

| **Confirmed cases managed on the day of the chemical attacks** | | | | |
| --- | --- | --- | --- | --- |
| **City** | **Dead** | **Affected** | **Total** | **Study** |
| **Matsumoto** | 7 | 272 | 279 ***** | Yanagisawa et al. (2006) ^9^ |
| **Tokyo** | 4 **^‡^** | 920 | 924 **^‡^** | Yanagisawa et al. (2006) ^9^; Novaki et al. (1995) ^6^ |
| **Damascus** | --- **^¥^** | 130 **^€^** | 130 **^€^** | Rosman et al. (2014) ^8^ |
|  |  | **Total** | **1333** |  |
|  |  |  |  |  |
| **Tokyo – Keio University, Emergency and Critical Care Department** | 1 **^‡^** | 100 (85+15 **^‡^**)**^†^** | 101 **^† ‡^** | Yanagisawa et al. (2006) ^9^; Novaki et al. (1995) ^6^ |
| **Tokyo – St. Luke’s Hospital** | 2 | 640 | 640 **^! ‡^** | Yanagisawa et al. (2006) ^9^; Okumura et al. (1996) ^7^ |

**Table S7. Calculation of cases of exposure to sarin gas per country and grand total.**

**Acronyms and Symbols**

***** Yanagisawa et al. (2006) reported seven (7) dead (5 at home, 2 at ER) and 264 exposed patients for a total of 279 cases managed in the Matsumoto area the day of the chemical attack (ER: 56; walk-in clinic: 208; medical staff affected secondarily: 8 individuals included among the ER or walk-in clinic counts) ^9^. If the 277 symptomatic individuals who did not seek medical attention were added to the 279 confirmed cases managed, the total would have been 556 ^9^.

**^†^** Yanagisawa et al. (2006) ^9^ and Novaki et al. (1995) ^6^ have little data in common regarding casualty management at Keio University ^6,9^. Novaki et al. (1995) reported medical staff treated 113 patients, 85 of which were managed on the day of the attacks ^6^. The authors did not provide further information about the 28 remaining patients’ whereabouts and health conditions ^6^. Also, as indicated by the authors’ method, 15 medical staff members were affected by contaminated patients ^6^. Therefore, 100 individuals were confirmed as cases affected by sarin gas the day of the attack (i.e.: 85 confirmed patients +15 medical staff members = 100 confirmed cases) ^6^. If the 28 cases where some uncertainty remains are added, the total would have been 128 affected individuals managed at Keio University the day of the attack ^6^. However, the count of 85 casualties at Keio University ^6,9^ appeared in both Yanagisawa et al. (1995)^9^ and Novaki et al. (1995) ^6^. Fifteen (15) medical members were reported by Novaki et al. (1995) as having been affected by sarin gas due to a secondary exposure resulting from the management of contaminated casualties ^6^. Yanagisawa et al. (1995) reported a similar number (15) related to admitted patients ^9^, but the authors presented no reference for that data ^9^. Novaki et al. (1995) also reported 15 exposed patients admitted at Keio ^6^. As this was supported by a reference (Suzuki et. al (1995) ^10^), these cases were thereafter not considered as relating to medical staff members ^6^. Both studies (Novaki et al. (1995); Yanagisawa et al. (2006)) were, therefore, not presenting duplicate data on affected medical members^6,9^. Yanagisawa et al. (2006) also reported an additional case dead on arrival at Keio ^9^. Therefore, the confirmed cases managed count at Keio was 101 rather than 100.

**^!^** Yanagisawa et al. (2006) ^9^ have little data in common with Okumara et al. (1996) ^7^ regarding casualty management at St. Luke’s ^7,9^. These shared data figures were the total number of casualties managed at St. Luke’s, which was 640 individuals the day of the attack ^7,9^. In Okumura et al. (1996), the two women that died as a result of the attack were included in the 640 managed victims ^7^. One died at her arrival at St. Luke’s while the other passed away 28 days after the chemical attack ^7^. Yanagisawa et al. (2006) reported the same information concerning the deceased women ^9^. Yanagisawa et al. (2006) also reported that 110 St. Luke’s medical staff were injured while treating 472 contaminated patients ^9^. Therefore, on the day of the attack, the confirmed number of cases managed by St. Luke’s was 1 dead and 749 affected (639 patients and 110 medical staff members), for a total of 750 cases ^9^. In order to avoid duplicate data, only statistics from Yanagisawa et al. (2006) were considered to arrive at the estimated grand total of confirmed cases managed at St. Luke’s ^9^.

**^‡^** The calculation for the confirmed number of cases managed in the Tokyo area the day of the attack was the sum of cases at the St. Luke’s, Keio and Teishin medical facilities. In addition, Yanagisawa et al. (2006) reported two (2) dead at the Tokyo train station ^9^. Based on the above information, duplication was prevented by excluding the 640 cases from Okumara and al. (1996) ^7^ and keeping those of Nozaki and al. (1995) only for their values concerning the medical staff affected and the one death ^6^. Thus, the grand total of confirmed cases managed in the Tokyo area was 924 (15 medical staff members (Keio; Nozaki and al. (1995)^6^) **plus** 909 remaining victims (Yanagisawa et al. (2006): i. St. Luke’s Hospital: 750 (1 dead and 749 affected individuals (639 patients and 110 medical staff members)); ii. Keio University: 85 patients and one (1) dead; iii. Teishin Hospital: 32 patients and 39 rescuer staff; iv. Subway station: 2 dead) ^9^**).** In other words, 920 individuals were affected and 4 died the day of the attack in the Tokyo area. By adding the 279 individuals affected in Matsumoto to the 924 in Tokyo, the grand total of confirmed cases managed the day of the two attacks in Japan was 1203 cases.

**^¥^** Rosman et al. (2014): In their analysis, the authors did not report deceased cases first-hand. They reported having obtained the data from the United Nations (ref) retrospectively.

**^€^** Rosman et al. (2014): Same data from Table S6.

Table S8 lists the definitions for triage used during the chemical attacks.

| **Study** | **Chemical Incident** | **Definition of Triage** | **Remarks** |
| --- | --- | --- | --- |
| Yanagisawa et al. (2006) ^9^ | Matsumoto (1994) | They defined degrees of severity terms as follows: i. severely affected – subjects admitted to hospital; ii. moderately affected – subjects who visited outpatient clinics; iii. slightly affected – subjects who reported having symptoms but did not seek medical attention |  |
|  | Tokyo (1995)  (St-Luke’s) | Patients were also graded according to the severity of their exposure, but no additional clinical information was presented (i.e.: symptomatology versus treatments) ^9^. These were: a. critically or severely injured: 5 people exhibited CPA or required mechanical ventilation; b. moderately injured: 107 people were characterized by systemic symptoms and signs of respiratory, digestive and/or neurological (central, peripheral or autonomic) systems in addition to ocular signs; c. mildly affected: 528 people only presented ocular signs or symptoms, and only required hospitalization for an hour before being discharged |  |
| Okumara et al. (1996) ^7^ | Tokyo (1995)  (St. Luke’s) | The 640 patients were graded according to severity levels which were defined and counted as follows: i. mild (528 patients or 82.5%): involving only ocular signs or symptoms (e.g. miosis, eye pain, dim vision, decreased visual acuity) on presentation, and hospital discharge after a 12-hour observation period at the ER; ii. moderate (107 patients or 16.7%): presence of systemic signs and symptoms (e.g. weakness, difficulty breathing, fasciculation, convulsions), but no mechanical ventilation requirements; iii. severe (five (5) or 78%): requiring emergency respiratory support (e.g. intubation and ventilation support) ^7^. In the first three hours, 106 patients received 2-PAM upon admission. In total, 111 patients with moderate to severe exposures (17.3%) were admitted to the ER while the remaining 529 were discharged shortly afterwards ^7^. | The entire casualty management effort at St. Luke’s the day of the attack, Okumara et al. (1996) reported some statistics on cases managed seen (i.e.: symptomatology versus treatments) ^7^ that are detailed differently than those found in Yanagisawa et al. (2014) ^9^. |

**Table S8. Algorithmic Definitions used for triage**.

**NOTE**. These definitions were reported without any reference.

**Acronyms. CPA** – Cardiac Pulmonary Arrest - **ER** – Emergency Room - **PAM** - pralidoxime iodide - **2-PAM** - 2‑pyridinealdoxime methiodide

**References**

1. Cochrane Guides and handbooks. Cochrane Training Website. http://training.cochrane. org/ handbooks. Version 6.2. Accessed (Last) August 1, 2021.

2. Celli BR, MacNee W, Agusti A, et al. Standards for the diagnosis and treatment of patients with COPD: a summary of the ATS/ERS position paper. *European Respiratory Journal*. 2004;23(6):932-946. doi:10.1183/09031936.04.00014304.

3. Pauwels RA, Buist AS, Calverley PMA, Jenkins CR, Hurd SS. Global Strategy for the Diagnosis, Management, and Prevention of Chronic Obstructive Pulmonary Disease NHLBI/WHO Global Initiative for Chronic Obstructive Lung Disease (GOLD) Workshop Summary. *Am J Respir Crit Care Med*. 2001;163:1256-1276.

4. Hong QN, Pluye P, Fàbregues S, et al. Improving the content validity of the mixed methods appraisal tool: a modified e-Delphi study. *Journal of Clinical Epidemiology*. 2019;111:49-59.e1. doi:10.1016/j.jclinepi.2019.03.008

5. Hawker S, Payne S, Kerr C, et al. Appraising the Evidence: Reviewing Disparate Data

Systematically. *Qualitative Health Research*. 2002;12(9):1284-1299. doi:10.1177/1049732302238251

6. Nozaki H, Hurl S, Shinozawa Y, et al. Secondary exposure of medical staff to sarin vapor

in the emergency room. *Intensive Care Med*. 1995;21:1032-1035

7. Okumura T, Takasu N, Ishimatsu S, et al. Report on 640 victims of the Tokyo subway sarin attack. *Ann Emerg Med*. 1996;28(2):129-135. doi:doi: 10.1111/j.1553-2712.1998.tb02470.x

8. Rosman Y, Eisenkraft A, Milk A, et al. Lessons learned from the Syrian sarin attack: evaluation of clinical syndrome through social media. *Ann Intern Med*. 2014;160(9):644-649.

9. Yanagisawa N, Morita H, Nakajima T. Sarin experiences in Japan: acute toxicity and long-term effects. *J Neurol Sci*. Nov 1 2006;249(1):76-85. doi:10.1016/j.jns.2006.06.007

10. Suzuki T, Morita H, Ono K, et al. Sarin poisoning in Tokyo subway. *The Lancet*. 1995;345(8955):980-981. doi:10.1016/s0140-6736(95)90726-2.
