# Supplementary material for: Gaps in Prehospital Care for Patients Exposed to a Chemical Attack – A Systematic Review
Source: Prehosp Disaster Med. 2022 Mar 11;37(2):230–9. doi: 10.1017/S1049023X22000401 (PMC8948487; doi:10.1017/S1049023X22000401)
Supplement: Supplementary file 1 [file S1049023X22000401sup.zip › S1049023X22000401sup004.docx]

# Supplemental Material

# Methods (Long Version)

*Study Design*

This study is a systematic review of the literature. The recommendations of the Cochrane Handbook for Systematic Reviews of Interventions were followed ^1^. The protocol was registered in the international prospective register for systematic reviews maintained by the National Institute for Health Research (PROSPERO, registration number: CRD42019104473, accepted on February 25, 2019, https://www.crd.york.ac.uk/prospero/; last update: November 24, 2020).

*Source of Data*

Online databases used for this study were: MEDLINE, Web of Science Core Collection, Embase, Cochrane, CINAHL, from their inception to November 6, 2018. An update was performed on September 16, 2020 (see Supplement, Table S1).

*Search Strategy*

In order to implement the search strategy and facilitate our analysis with terms describing contextual circumstances, we defined two categories of warfare. After consulting the International Security Center at Laval University, we concluded that contrary to medical concepts like chronic obstructive pulmonary disease (COPD) ^2,3^, there were no gold-standard terms defining conventional and asymmetric warfare (including terrorism and criminal activities). Thus, conventional warfare was defined as armed conflicts opposing states where political authorities employ their respective military capability within the international law of armed conflict framework. Asymmetrical warfare, on the other hand, involves rogue states or organizations seeking to gain a political advantage using diverse means (e.g.: military, cyber, psyops, finance, terrorism, criminality, etc.), usually accompanied by the use of force or violence.

Indexed and free-text terms, such as Respiratory, Warfare and Chemical Threat were selected by individually combining each of the two warfare modes with respiratory distress (See Table S1). Afterwards, references were imported into the Covidence systematic review software (Veritas Health Innovation, Melbourne, Australia, available at www.covidence.org). Duplicate papers were automatically rejected by this software.

*Research Staff Structure and Functioning*

Two triage sequences involving two-member teams were designed for this study (first triage: titles and/or abstracts of studies; second triage: full-text screening, based on the inclusion and exclusion criteria). For the quality appraisal and data extraction sequences, the same staff composition was used. All personnel received Cochrane training provided by Cochrane-Francophone (Quebec, Canada) or by their home university. Additional training was provided on chemical, biological, radiological, nuclear, explosive (CBRNE) defence for those with no professional background in this specialist field. A triage performance test consisting in the evaluation of forty papers was administered to all triage staff.

Each team member worked independently of the other, making his or her own determination directly in the Covidence software based on the inclusion and exclusion criteria. If disagreements occurred at any point in the above-mentioned process, a consensus approach was initially applied between staffers. If this failed to resolve the issue, an independent resolution panel comprised of two senior staff members, a senior medical officer and a researcher, decided on the eligibility of the paper in dispute. At the end, member determinations differed less than 10% of the time.

*Inclusion and Exclusion Criteria*

Inclusion criteria were: i. exposure to a chemical incident (e.g.: mass casualties); ii. chemical known to affect the respiratory system; iii. interventions involving the assessment of a triad of integrated key competences (1. protection for staff and patients, 2. decontamination (immediate and specialized) and 3. treatments iv. patient outcomes (i.e.: primary: patient’s health condition remaining stable due to medical, protection and decontamination interventions; secondary: patient’s mortality occurring at his/her admission despite medical, protection and decontamination interventions); v. studies with original data, including those conducted on animals induced with chemical agents in order to simulate a medical extraction of casualties; vi studies should have occurred in our zone of interest. The zone of interest where medical interventions took place in eligible studies was defined as the casualty extraction from the incident site where the chemical attack occurred to the clean zone, where the patient was admitted to the hospital (Figure 1).

Studies were excluded if: i. effects were shown on insects, plants or materials; ii. procedures were performed in a clean/cold zone setting once the patient is fully admitted and handled by the medical facility’s staff; iii. they did not address a respiratory disorder; iv. it they did not present original data (e.g.: reviews); v. the topic was not related to a chemical threat (e.g.: suicide attempt).

*Quality Appraisal/Risk of Bias*

Two quality appraisal charts were used in order to detect and mitigate the variability in staffers’ assessments. The first was developed by Hong et al. (2018) from McGill University ^4^. The second was from Hawker et al. (2002) (Appendices C and D) ^5^. The risk of bias in each eligible study was assessed independently by two reviewers.

*Data Extraction*

Due to the complexity of intervening in environments contaminated by chemicals, biologicals, or ionizing radiation (i.e.: radiological and nuclear), criteria for the extraction sheet were tailored to this field in order to optimize the analysis.

*Synthesis of Evidence*

Evidence from the eligible studies was summarized according to: i. algorithms used to respond to a chemical attack (i.e.: from disaster management plans to early-warning systems used to respond to vital distress; ii. detection of clinical signs indicating the chemical agent affected the patient’s condition (e.g.: stabilization, health deterioration, death, etc.) along with clinical interventions (including treatment, protection and decontamination); iii. time elapsed until the start of the clinical response, including protection and decontamination procedures; iv. association between the above-mentioned variables.

*Biostatistical Analysis*

Descriptive statistics were planned to summarize study characteristics, including mean and standard deviations, median and interquartile range and proportions, according to the type of data. A Student’s t-test was planned to compare the clinical onset of chemical agents and algorithms of treatment, along with forest plots to highlight the difference between each agent’s action mechanism and therapy onsets. To mitigate the potential impact of missing data, an imputation model was planned (root mean square error). Descriptive statistics and other numbers were to be computed with IBM SPSS Statistics Software (SPSS Inc., Chicago, IL, USA) and StatsDirect statistical software, StatsDirect Ltd. Sale, Cheshire, UK). A meta-analysis involving the use of a random effects linear model (mixed effects model) was planned to correlate the effect of a studied chemical agent with one of the clinical interventions made by health care professionals. This would have highlighted the windows of treatment opportunities in such contaminated environments (RevMan software version 5.3, The Cochrane Collaboration Network, London, United Kingdom). The statistical significance level was set at p<0.05 and interpreted with 95% confidence intervals. Our biostatistics plan had been reviewed by a biostatistician. Unfortunately, it was not possible to run any statistical analysis due to the heterogeneity of eligible studies and paucity of extractable data.

# Additional Results

*Languages other than English*

Among the studies identified by the initial search strategy, a total of 32 papers were published in a language other than English. After a full-translation or based on the English abstract, these studies were excluded as they did not meet eligibility criteria. The language breakdown is as follows: i. Japanese (7x); ii. German (5x); iii. Chinese (4x); iv. Czech (3x); v. Dutch (2x); vi. Russian (2x); vii. Turkish (2x); viii. Danish (1x); ix. Greek (1x); x. Hebrew (1x); xi. Italian (1x); xii. Persian (1x); xiii. Polish (1x); xiv. Spanish (1x).

*Medical Interventions Surrounding Patient Admission to Medical Facilities*

Some of the interventions performed after patients were admitted to medical facilities were reported as continuity of care ^6-9^. In Nozaki et al. (1995), the authors reported that cardiac resuscitation manoeuvres were limited to 40 minutes in duration, but provided no details on the management of their convulsive patients ^6^. There was also no medical interventions reported on the 83 remaining contaminated patients that the medical staff continued to treat ^6^. Moreover, the treatment listing on chemical incidents in Japan (See Table 2) did not include oxygen therapy.

*Other Secondary Exposure Causes*

While three ^7-9^ of the four ^6-9^ studies did not fully cover secondary exposures, they described occurrences of close contacts between unexposed clinicians/rescuers and contaminated patients. Of the 640 cases reported in Okumara et al. (1996), the medical staff had to manage 106 described as moderate-to-severe within the first three hours after the attack ^7^; including five (5) classified as severe ^7^. The authors reported all 106 patients received 2-PAM, diazepam and other medications within that timeframe ^7^, but did not address exposure duration in their study ^7^. Yanagisawa et al. (2006) ^9^ provided some information on the transport of contaminated patients and their management across unspecified hospitals in the Matsumoto area in 1994 ^7^. The authors mentioned that the rescuers and medical staff were not aware that sarin was the cause of the incident ^7^. They also reported that one of the eight medical staff who became affected spent five hours in the contaminated incident zone ^7^. The first patients reached the hospital nearly six hours after the attack ^7^. Of the three cardiac arrest cases managed by the staff at the ER, two died within four hours of their admission/observation ^7^. As was the case in the Tokyo attack in 1995, Yanagisawa et al. (2006) ^9^ provided information demonstrating closer proximity between clinicians, rescuer teams and medical staff as a result of their interventions (Keio University Hospital: patient admission processes, serology testing, drug injection; Teishin hospital: patient transportation and admission; St. Luke’s: 110 medical staff members affected while treating 417 patients) ^9^. The authors reported that sarin had been identified as the cause of intoxications two hours after the attack ^9^. Rosman et al. (2014), for their part, enumerated treatments provided, such as access to parenteral routes for drug injection and airway management for vital assistance ^8^.

*Disaster Plan*

Okumura et al. (1996) was the only paper to confirm the use of a disaster plan ^7^. In the case of Nozaki et al. (1995), the medical staff were notified of a gas explosion in the Tokyo subway and the transfer of casualties by the fire agency ^6^. No further detail was provided in both studies.

*Telemedicine*

Rosman et al. (2014) was the only study that conducted a retrospective analysis of YouTube footage of chemical attacks that had just occurred ^8^. However, as reported by the authors themselves, the authenticity of the videos remains uncorroborated ^8^ .

*Security Intelligence*

None of the four studies ^6-9^ conducted a comprehensive effect analysis related to the deployment of the chemical agents and of various factors such as the weather, terrain, means of delivery and *modus operandi* aimed at causing death and injury in a densely populated area.

# Additional Discussion

*Social Media as a Potential Emergent Capability in the Clinical Approach*

Interestingly, one study analyzed YouTube videos of the 2013 Syrian chemical attack despite the authors casting doubts on the authenticity of the footage ^8^. To our knowledge, there have been no similar studies reported in the health science literature. However, social media, along with live streaming and artificial intelligence capabilities, may represent an opportunity to improve care for mass casualties in the context of a chemical attack due to terrorism or warfare.

*Importance of Data Gathering in a Chaotic Mass Casualty Context*

In all four published papers ^6-9^, no information is provided regarding the existence of pre-hospital data gathering systems for monitoring and recording interventions. There was also a lack of epidemiology, notably in infants, the elderly, women and populations suffering from chronic diseases ^6-9^. This highlighted the quasi-blind eye turned, by default, on the gathering of evidence necessary to understand chemical exposures thoroughly. Since the Matsumoto and Tokyo attacks in 1994-1995, and the 2014 attack in Syria, digital medical information systems have evolved significantly and will continue to do so. By incorporating artificial intelligence, such systems will in future be capable of processing mega-data in real time, and of implementing automated commands. They may require little infrastructure and reduce the burden on the clinician, particularly in complex situations. On the following website, this current study proposes such a case report form integrated into a clinical cohort study for chemical attacks approved by an ethical committee (https://rsr-qc.ca/en/ltb/; last accessed: 14 June 2021). However, such an approach requires specific resources dedicated to data collection. Finally, other types of incidents such as the biological, radiological and nuclear kind should also be investigated.

*Importance of Pursuing Research in Acute Settings*

Despite the lack of coverage of acute settings in the literature as demonstrated by the publication of only four post-incident papers, there is still a need to further pursue this research. Progress in other scientific fields, including animal models, leads to envision clinical research directly involving decontamination and protection capabilities under the same umbrella.
