# Supplementary material for: Gaps in Prehospital Care for Patients Exposed to a Chemical Attack – A Systematic Review
Source: Prehosp Disaster Med. 2022 Mar 11;37(2):230–9. doi: 10.1017/S1049023X22000401 (PMC8948487; doi:10.1017/S1049023X22000401)
Supplement: Supplementary file 1 [file S1049023X22000401sup.zip › S1049023X22000401sup002.docx]

| **Serial number** | **Questions** | **Answers found in the articles** | **Comments or clarifications** |
| --- | --- | --- | --- |
| **1** | **Identification – Article** | | |
| **2** | Citation, APA style | N/A |  |
| **3** | **Study details** | | |
| **4** | Sponsor |  |  |
| **5** | Country |  |  |
| **6** | Setting |  |  |
| **7** | **Author’s contact information** | | |
| **8** | Author’s name |  |  |
| **9** | Institution |  |  |
| **10** | Email |  |  |
| **11** | Address |  |  |
| **12** | **Identification – Threat** | | |
| **13** | **Geopolitical target** | | |
| **14** | Country |  |  |
| **15** | Population of country |  |  |
| **16** | City |  |  |
| **17** | Population of city |  |  |
| **18** | **Geopolitical motivation** | | |
| **19** | Did the threat occur in wartime or other military operations? |  |  |
| **20** | Did the threat occur in peacetime? |  |  |
| **21** | Did the threat occur during or after the Cold War against high-value targets (e.g.: Intelligence operation)? |  |  |
| **22** | Was the chemical threat known at the moment of the attack? |  |  |
| **23** | Did the chemical threat occur during a surprise attack? |  |  |
| **24** | What were the motives behind the attack? |  |  |
| **25** | When was the chemical attack confirmed? (timeframe) |  |  |
| **26** | **Chemical agent(s)** | | |
| **27** | Was the chemical agent known at the time of the attack? |  |  |
| **28** | When was the chemical agent confirmed? (timeframe) |  |  |
| **29** | What was the chemical agent? |  |  |
| **30** | **Affected population characteristics** | | |
| **31** | **In total** | | |
| **32** | Total number of casualties |  |  |
| **33** | **People treated in this study** | | |
| **34** | How many people were treated in this study? |  |  |
| **35** | Gender |  |  |
| **36** | Age at the time of the event |  |  |
| **37** | Race |  |  |
| **38** | Health status prior the incident |  |  |
| **39** | **Characteristics of pre-hospital resources** | | |
| **40** | Number of paramedics working as staff in the attack area |  |  |
| **41** | How many ambulances were deployed? |  |  |
| **42** | Were all paramedics qualified to administer drugs and perform life-support maneuvers on exposed patients? |  |  |
| **43** | How many paramedics were allowed to administer drugs and perform life-support maneuvers on exposed patients? |  |  |
| **44** | Were the paramedics and their deployed assets military? |  |  |
| **45** | **Characteristics of health care facilities** | | |
| **46** | What was the name of the hospital? |  |  |
| **47** | Usual number of admissions via the ER each year |  |  |
| **48** | Number of physicians working as staff in the ER |  |  |
| **49** | Number of nurses working as staff in the ER |  |  |
| **50** | Number of paramedics working as staff in the ER |  |  |
| **51** | Number of respiratory therapists working as staff in the ER |  |  |
| **52** | Has a Disaster Mass-Casualty Plan ever been used? |  |  |
| **53** | Did that include CBRNE events? |  |  |
| **54** | Was the work-rest cycle applied successfully during the chemical incident? |  |  |
| **55** | **Actions right after the attack** | | |
| **56** | Was first aid administered? |  |  |
| **57** | If yes, specify within what time interval |  |  |
| **58** | Were medical treatments administered right after the attack by nearby medical resources? |  |  |
| **59** | If yes, specify what type of medical resources |  |  |
| **60** | If yes, specify within what time interval |  |  |
| **61** | **Medical response and extraction** | | |
| **62** | By which means of transportation did the patient(s) arrive at the hospital? |  |  |
| **63** | Within which time intervals did the medical responders arrive? |  |  |
| **64** | Within which time intervals did the exposed patient present first signs and symptoms? |  |  |
| **65** | Within which time intervals were the first treatments initiated? |  |  |
| **66** | What were the interventions performed during transportation to the hospital? |  |  |
| **67** | **Protection** | | |
| **68** | What type of individual protective equipment did personnel wear? (Gas mask, protective suit, gloves and boots, etc.) |  |  |
| **69** | What type of individual protective equipment did your patient wear? (Gas mask, protective suit, gloves and boots, etc.) |  |  |
| **70** | **Immediate Decontamination** | | |
| **71** | As is normally the case in military operations, were health-care personnel and patients immediately decontaminated? |  |  |
| **72** | **Decontamination (DECON)** | | |
| **73** | Was a decontamination facility deployed? |  |  |
| **74** | Did the decontamination set-up have a clean zone? |  |  |
| **75** | Where did the decontamination assets deploy? |  |  |
| **76** | Was a decontaminant used? |  |  |
| **77** | Was the decontaminant specific for treating an exposed casualty? |  |  |
| **78** | Did the patients wear a respiratory protective device during the DECON process? |  |  |
| **79** | If so, did the patient wear a gas mask? |  |  |
| **80** | If yes, was the patient’s mask removed right after the decontamination? |  |  |
| **81** | Was the patient’s skin brushed during the decontamination? |  |  |
| **82** | **Methods** | | |
| **83** | **Design** | | |
| **84** | What was the type of study design? |  |  |
| **85** | What was the model used (e.g.: animal, human subject, tissue, mannequin, bench, etc.)? |  |  |
| **86** | Was any type of study enrolment conducted (including narrowing sample groups)? |  |  |
| **87** | What were the inclusion and exclusion criteria? |  |  |
| **88** | Was the study a Phase I clinical trial? |  |  |
| **89** | Was randomization used? |  |  |
| **90** | Were blind means used for the investigator or the subjects? |  |  |
| **91** | How many arms did the study have? |  |  |
| **92** | Did the study have a control and/or placebo group? |  |  |
| **93** | If so, what were their specific criteria (e.g.: age, race, etc.)? |  |  |
| **94** | Did the study have any prerequisites (i.e.: ethical approval, patient approval, medical condition(s) other than having been exposed to chemical weapons, medical examination and tests)? |  |  |
| **95** |  |  |  |
| **96** | **Measurement(s)** | | |
| **97** | What was the main measurement in this study? |  |  |
| **98** | What were the other measurements involved in this study (list concisely)? |  |  |
| **99** | Was there any level of judgment criteria? |  |  |
| **100** | What were the means of data gathering used? |  |  |
| **101** | What was the statistics plan used? |  |  |
| **102** | What were the data analysis plan used (including: biostatistics) |  |  |
| **103** | For biostatistics, what was the significant threshold p-value? |  |  |
| **104** | If any measurement used required any kind of validation (including calibration of instruments)? |  |  |
| **105** |  |  |  |
| **106** | **Results (see first paragraph of the discussion and conclusion sections as they usually provide a concise version of the main findings)** | | |
| **107** | What were the main results (particularly those with biostatistical significance)? |  |  |
| **108** | Was any statistical inference reported in this study? |  |  |
| **109** | What were the secondary findings (less relevant but reported)? |  |  |
| **110** | Did the author(s) report any study limitations and biases? |  |  |
| **111** | Were any of the following compromised and what were the mitigation means used? (Data, models, means of measurement) |  |  |
| **112** | Did the author(s) report the findings as clinically relevant or not? |  |  |
| **113** | What were the relevant clinical data (i.e.: signs and symptoms, including treatments) applicable to our research questions, hypothesis, objectives or centre of interest? |  |  |
| **114** | In terms of other relevant clinical data (i.e.: signs and symptoms, including treatments), what are they in summary? |  |  |
| **115** | **Discussion (here to be summarized)** | | |
| **116** | What were the key points of the discussion? |  |  |
| **117** | What points were stressed/highlighted by the paper? |  |  |
| **118** | What were the recommendations, if any? |  |  |
| **119** | What were the study limitations, if any? |  |  |
| **120** | Is there a fact or data significant enough to be extracted (e.g.: new SpO2 target levels 88–92%)? |  |  |
| **121** |  |  |  |
| **122** | **Complex outcomes** | | |
| **123** | Alive (Specify within which timeframe) |  |  |
| **124** | Deceased (specify within which timeframe) |  |  |
| **125** |  |  |  |
| **126** |  |  |  |
| **127** | **Conclusion** | | |
| **128** | Was any future perspective or recommendation mentioned? |  |  |
| **129** |  |  |  |
| **130** | **Discussion, Tables, Figures, Equation and References (Here we seek quantitatively and out of order statement(s)** | | |
| **131** | Was there anything special reported in the discussion? |  |  |
| **132** | How many tables did the study have (including supplements)? |  |  |
| **133** | How many figures did the study have (including supplements)? |  |  |
| **134** | How many equations did the study have (including supplements)? |  |  |
| **135** | How many videos did the study have (including supplements)? |  |  |

**Table S2 – Extraction Sheet Template**

NOTE. Instructions: Find the answers in the article. Do not speculate or try to guess the answers. If an answer is not found in the article, write unspecified. If you want to add something, use the comments or clarifications column.
