# Supplementary material for: Gaps in Prehospital Care for Patients Exposed to a Chemical Attack – A Systematic Review
Source: Prehosp Disaster Med. 2022 Mar 11;37(2):230–9. doi: 10.1017/S1049023X22000401 (PMC8948487; doi:10.1017/S1049023X22000401)
Supplement: Supplementary file 1 [file S1049023X22000401sup.zip › S1049023X22000401sup001.docx]

| **MEDLINE (PubMed) – 686** | |
| --- | --- |
| **Respiratory**  (1622390) | "Respiration Disorders"[Mesh]  OR "Respiratory Mucosa"[Mesh]  OR "Respiratory Physiological Phenomena"[Mesh]  OR Respirat*[TIAB] OR Pulmonary [TIAB]  OR "Airway Remodeling"[TIAB]  OR "work of breathing"[TIAB] OR "breathing work"[TIAB] OR "breathing works"[TIAB]  OR "Airway Resistance"[TIAB]  OR "Lung Compliance"[TIAB]  OR "Mucociliary Clearance"[TIAB]  OR "Respiratory Muscles"[Mesh]  OR "Bronchioles"[Mesh] OR bronchiole*[TIAB]  OR "Signs and Symptoms, Respiratory"[Mesh]  OR "Airway Management"[Mesh]  OR "Vital Signs"[Mesh]  OR "Vital Sign"[TIAB] OR "Vital Signs"[TIAB]  OR "Thoracic Wall"[Mesh]  OR "Thoracic Wall"[TIAB]  OR "Chest Wall"[TIAB] OR "Death, Sudden"[Mesh] OR "Sudden Death"[TIAB]  OR "Burns, Inhalation"[Mesh] OR "Inhalation Burn"[TIAB] OR "Inhalation Burns"[TIAB]  OR "respiratory acidosis"[TIAB] OR "Acidosis, Respiratory"[Mesh]  OR "tidal volume"[TIAB] OR "tidal volumes"[TIAB] OR "Lung Volume Measurements"[Mesh]  OR "Lung Capacity"[TIAB] OR "Lung Capacities"[TIAB] OR "Lung Volume"[TIAB]  OR "diaphragmatic paralysis"[TIAB] |
| **Warfare**  (64954) | "Chemical Warfare"[Mesh] OR "Chemical Warfare"[TIAB] OR "Chemical Warfare"[TIAB]  OR "Biological Warfare Agents"[Mesh] OR "Biological Warfare"[Mesh]  OR Bioterror* [TIAB] OR bio-terror*[TIAB] OR "Biological Weapon"[TIAB] OR "Biological Weapons"[TIAB]  OR "biological attack"[TIAB] OR "biological attacks"[TIAB]  OR "Chemical Warfare Agents"[Mesh]  OR "Weapons of Mass Destruction"[Mesh:NoExp]  OR "Weapons of Mass Destruction"[TIAB] OR "Weapon of Mass Destruction"[TIAB]  OR "Warfare Agent"[TIAB] OR "Warfare Agents"[TIAB]  OR "Biothreat Agent"[TIAB] OR "Biothreat Agents"[TIAB]  OR "Biological warfare"[TIAB]  OR "terrorist attack"[TIAB] OR "terrorist attacks"[TIAB]  OR Terrorism[TIAB]  OR "Asymmetric War"[TIAB]  OR "conventional war"[TIAB] OR "conventional wars"[TIAB]  OR "Non-conventional war"[TIAB] OR "Non-conventional wars"[TIAB]  OR peace-keeping[TIAB] OR peacekeeping[TIAB]  OR military[TIAB]  OR "political assassination"[TIAB] OR "political assassinations"[TIAB]  OR "intelligence operation"[TIAB] OR "intelligence operations"[TIAB]  OR "mass casualties"[TIAB] OR "mass casualty"[TIAB] |
| **Chemical Threat**  (1126508) | "Chemical Hazard Release"[Mesh]  OR "Biohazard Release"[Mesh]  OR "chemical hazard release"[TIAB] OR "biohazard release"[TIAB]  OR "Nerve Agents"[Mesh] OR "Nerve Agent"[TIAB] OR "Nerve Agents"[TIAB] OR "nerve gas"[TIAB]  OR "Choking agent"[TIAB] OR "Choking agents"[TIAB]  OR "Blister agent"[TIAB] OR "Blister agents"[TIAB]  OR "blood agent"[TIAB] OR "blood agents"[TIAB] OR "suffocating agent"[TIAB] OR "suffocating agents"[TIAB]  OR "neurotoxic agent"[TIAB] OR "neurotoxic agents"[TIAB]  OR tabun [Supplementary Concept] OR tabun[TIAB]  OR "Sarin"[Mesh] OR sarin[TIAB]  OR "Soman"[Mesh] OR soman[TIAB]  OR "VX" [Supplementary Concept] OR "agent VX"[TIAB]  OR "Mustard Gas"[Mesh] OR "mustard gas"[TIAB]  OR "S-176 mustard" [Supplementary Concept] OR "s-mustard"[TIAB] OR "n-mustard"[TIAB]  OR "lewisite" [Supplementary Concept] OR lewisite[TIAB]  OR "Chemical Warfare Agents" [Pharmacological Action]  OR "trichloromethyl chloroformate" [Supplementary Concept] OR "trichloromethyl chloroformate" [TIAB] OR diphosgene[TIAB] OR chloropilkrin[TIAB]  OR "Hydrogen Cyanide"[Mesh] OR Cyanide[TIAB]  OR Ammonia[Mesh] OR ammonia[TIAB]  OR "Carbon Tetrachloride Poisoning"[Mesh] OR "Carbon Tetrachloride"[Mesh] OR "carbon tetrachloride"[TIAB]  OR "Chlorides"[Mesh] OR Chloride*[TIAB]  OR "hydrogen-chloride symporter"[Supplementary Concept]  OR "Hydrogen Sulfide"[Mesh] OR "Hydrogen Sulfide"[TIAB]  OR Methylamine[TIAB] OR "methylamine"[Supplementary Concept]  OR "Sulfur Dioxide"[Mesh] OR "Sulfur Dioxide"[TIAB]  OR "Phosgene"[Mesh] OR Phosgene[TIAB]  OR "phosphine"[Supplementary Concept] OR phosphine[TIAB]  OR "Dioxins"[Mesh] OR "Dioxins and Dioxin-like Compounds"[Mesh] OR Dioxin*[TIAB]  OR "cyanogen chloride"[Supplementary Concept]  OR "Cyclohexanes"[Mesh] OR Cyclohexanes[TIAB]  OR "omega-Chloroacetophenone"[Mesh] OR Chloroacetophenone[TIAB]  OR "o-Chlorobenzylidenemalonitrile"[Mesh] OR Chlorobenzylidenemalonitrile[TIAB]  OR hemotoxic[TIAB]  OR Vesicant*[TIAB]  OR Napalm[TIAB]  OR "Herbicides"[Pharmacological Action]  OR "Herbicides"[Mesh] OR Herbicide*[TIAB]  OR "Agent Orange"[Mesh] OR "agent orange"[TIAB]  OR "Phosphorus"[Mesh] OR "phosphorus chloride"[Supplementary Concept] OR "Phosphorus Compounds"[Mesh] OR Phosphorus[TIAB]  OR "Toxins, Biological"[Mesh] OR "biological toxin"[TIAB] OR "biological toxins"[TIAB]  OR "Ricin"[Mesh] OR ricin[TIAB]  OR botuli*[TIAB] OR "clostri-perfringens toxin"[TIAB] OR mycotoxin[TIAB] OR palytoxin*[TIAB] OR saxitoxin*[TIAB] OR "Staphylococcus enterotoxin"[TIAB] OR tetrodoxin[TIAB]  OR "Hydrocarbons, Halogenated"[Mesh: NoExp] OR "halogenated hydrocarbons"[TIAB] OR "halogenated hydrocarbon"[TIAB]  OR Thermine [TIAB] OR Formaldehyde[TIAB] OR "Formaldehyde"[Mesh]  OR Oxime*[TIAB] OR "Oximes"[Mesh]  OR "SEB intoxication"[TIAB] |
| **Embase (embase.com) - 979** | |
| **Respiratory** (1,818,402) | 'respiratory function disorder'/exp OR 'respiratory mucosa'/exp OR 'respiratory function'/exp  OR Respirat*:ti,ab,kw OR Pulmonary:ti,ab,kw  OR 'airway remodeling'/exp OR 'airway resistance'/exp  OR (Airway NEAR/2 (Remodeling OR Resistance)):ti,ab  OR "mucociliary clearance":de,ti,ab,kw  OR "lung compliance":de,ti,ab,kw  OR 'respiratory function disorder'/exp  OR "diaphragm paralysis":de,ti,ab,kw  OR "breathing muscle":de OR "breathing muscle*":ti,ab,kw  OR "bronchiole":de OR bronchiole*:ti,ab,kw  OR "breathing mechanics":de OR "breathing mechanic*":ti,ab,kw  OR "respiration control":de,ti,ab,kw  OR "Airway Management":ti,ab,kw  OR 'vital sign':de,ti,ab,kw OR "Vital Sign*":ti,ab,kw  OR "thorax wall':de,ti,ab,kw OR "Chest Wall":ti,ab,kw  OR "sudden death":de,ti,ab,kw  OR 'lung burn'/exp OR (burn* NEAR/2 (inhalation OR pulmonary OR lung*)):ti,ab  OR 'lung volume'/exp  OR ((tidal OR lung*) NEAR/2 (volume OR capacit*)):ti,ab  OR "work of breathing":de,ti,ab,kw OR "breathing work*":ti,ab,kw |
| **Warfare**  (106,389) | 'chemical warfare'/exp OR 'terrorism'/exp OR 'military phenomena'/de OR 'army'/de OR 'military research'/de OR 'war'/exp OR 'biological warfare'/exp  OR "weapon of mass destruction":de  OR ((Biological OR chemical OR "mass destruction") NEAR/2 (Weapon* OR warfare)):ti,ab  OR ((Warfare OR Biothreat) NEAR/2 Agent*):ti,ab  OR ("terrorist* attack*" OR Terrorism OR bio-terror* OR bioterror* OR peace-keeping OR peacekeeping OR military):ti,ab,kw  OR ((Asymmetric OR conventional OR Non-conventional) NEAR/2 war*):ti,ab  OR "political assassination*":ti,ab,kw  OR "intelligence operation*":ti,ab,kw  OR "mass casualt*":ti,ab,kw |
| **Chemical Threat**  (1,301,038) | 'chemical accident'/de OR 'biological accident'/de OR "Chemical Hazard Release":ti,ab,kw OR "biohazard release":ti,ab,kw  (Nerve NEAR/2 (Agent* OR gas)):ti,ab  OR "blood agent*":ti,ab,kw  OR "Choking agent*":ti,ab,kw  OR "Blister agent*":ti,ab,kw OR hemotoxic:ti,ab,kw  OR "suffocating agent*":ti,ab,kw  OR "neurotoxic agent*":ti,ab,kw OR 'neurotoxin'/exp  OR tabun:ti,ab,kw OR sarin:ti,ab,kw OR soman:ti,ab,kw  OR "agent VX":ti,ab,kw  OR 'toxic gas'/exp OR "mustard gas":ti,ab,kw OR "s-mustard":ti,ab,kw OR "n-mustard":ti,ab,kw  OR Phosgene:ti,ab,kw OR "hydrogen sulfide":ti,ab,kw  OR lewisite:ti,ab,kw  OR "trichloromethyl chloroformate":ti,ab,kw OR diphosgene:ti,ab,kw OR chloropilkrin:ti,ab,kw  OR 'cyanide':de OR cyanide*:ti,ab,kw  OR 'ammonia':de,ti,ab,kw  OR "carbon tetrachloride":de,ti,ab,kw  OR 'chloride'/de OR 'chloride*':ti,ab,kw OR 'hydrochloric acid'/de  OR "methylamine":de,ti,ab,kw  OR "sulfur dioxide":de,ti,ab,kw  OR "phosphine":de,ti,ab,kw  OR dioxin:de OR dioxin*:ti,ab,kw  OR 'cyanogen chloride':de  OR "cyclohexane derivative":de OR Cyclohexane*:ti,ab,kw  OR "phenacyl chloride":de OR Chloroacetophenone:ti,ab,kw  OR "2 chlorobenzylidenemalononitrile":de OR Chlorobenzylidenemalonitrile:ti,ab,kw  OR Vesicant*:ti,ab,kw OR Napalm:ti,ab,kw  OR 'herbicide'/exp OR herbicide*:ti,ab,kw  OR '2,4,5 trichlorophenoxyacetic acid'/de OR "Agent Orange":ti,ab,kw  OR "phosphorus":de,ti,ab,kw OR 'phosphorus derivative'/de  OR 'toxin'/exp  OR 'ricin':de,ti,ab,kw  OR "clostri-perfringens toxin*":ti,ab,kw OR mycotoxin*:ti,ab,kw OR palytoxin*:ti,ab,kw OR saxitoxin*:ti,ab,kw OR "Staphylococcus enterotoxin*":ti,ab,kw OR tetrodoxin*:ti,ab,kw OR botuli*:ti,ab,kw  OR 'halogenated hydrocarbon'/de OR "halogenated hydrocarbon*":ti,ab,kw  OR Thermine:ti,ab,kw  OR 'formaldehyde':de OR Formaldehyde:ti,ab,kw  OR oxime:de OR Oxime*:ti,ab,kw  OR "SEB intoxicat*":ti,ab,kw |
| **Web of Science Core Collection- 373** | |
| **Respiratory** (1,013,113) | TS= (Respirat* OR Pulmonary  OR (Airway NEAR/2 (Remodeling OR Resistance OR Management))  OR Bronchiole*  OR "Mucociliary Clearance"  OR "Vital Sign*"  OR "Sudden Death"  OR (Wall NEAR/2 (Thoracic OR Chest))  OR "Inhalation Burn*"  OR "tidal volume*"  OR "diaphragm paralysis"  OR "work of breathing" OR "breathing work*"  OR (lung* NEAR/2 (Capacit* OR Volume OR Compliance))) |
| **Warfare**  (168,522) | TS= (((Biological OR chemical OR "mass destruction") NEAR/2 (Weapon* OR warfare))  OR ((Warfare OR Biothreat) NEAR/2 Agent*)  OR "terrorist* attack*" OR Terrorism OR bioterror* OR bio-terror* OR peace-keeping OR peacekeeping OR military  OR ((Asymmetric OR conventional OR "Non-conventional") NEAR/2 war*)  OR "political assassination*"  OR "intelligence operation*"  OR "mass casualt*") |
| **Chemical Threat**  (1,218,341) | TS= ("Chemical Hazard Release" OR "biohazard release"  OR (Nerve NEAR/2 (Agent* OR gas))  OR "blood agent*" OR "choking agent*" OR "blister agent*" OR "suffocating agent*" OR "neurotoxic agent*" OR hemotoxic  OR tabun OR sarin OR soman OR "agent VX" OR "toxic gas" OR "mustard gas" OR "s-mustard" OR "n-mustard" OR Phosgene OR lewisite OR "hydrogen sulfide" OR cyanide* OR "trichloromethyl chloroformate" OR disphogene OR chloropilkrin  OR ammonia  OR "carbon tetrachloride"  OR chloride*  OR methylamine  OR "sulfur dioxide"  OR phosphine  OR dioxin*  OR cyclohexane  OR Chloroacetophenone  OR Chlorobenzylidenemalonitrile  OR Vesicant* OR Napalm  OR herbicide* OR "Agent Orange"  OR phosphorus  OR toxin*  OR ricin  OR "clostri-perfringens toxin*"  OR mycotoxin*  OR palytoxin*  OR saxitoxin*  OR "staphylococcus enterotoxin*" OR tetrodoxin* OR botuli*  OR "halogenated hydrocarbon"  OR Thermine  OR Formaldehyde  OR Oxime*  OR "SEB intoxicat*") |
| **CINAHL Plus With Full Text (EBSCO) - 53** | |
| **Respiratory**  (152,403) | MH "Respiration Disorders+"  OR MH "Respiratory Mucosa+"  OR MH "Respiratory Tract Physiology+"  OR MH "Respiratory Muscles+"  OR MH "Bronchioles"  OR MH "Respiratory Mechanics+"  OR MH "Signs and Symptoms, Respiratory"  OR MH "Airway Management+"  OR MH "Vital Signs+"  OR MH "Death, Sudden+"  OR MH "Burns, Inhalation+"  OR MH "Lung Volume Measurements+"  OR MH "Vital Capacity+"  OR MH "Mucociliary Clearance"  OR MH "Work of Breathing"  OR TI (Respirat*OR Pulmonary) OR AB (Respirat*OR Pulmonary)  OR TI (Airway N2 (Remodeling OR Resistance OR management)) OR AB (Airway N2 (Remodeling OR Resistance OR management))  OR TI "lung compliance" OR AB "lung compliance"  OR TI "mucociliary clearance" OR AB "mucociliary clearance"  OR TI "diaphragm paralysis" OR AB "diaphragm paralysis"  OR TI "vital sign*" OR AB "vital sign*"  OR TI bronchiole* OR AB bronchiole*  OR TI (Wall N2 (Thoracic OR Chest)) OR AB (Wall N2 (Thoracic OR Chest))  OR TI ((tidal OR lung*) N2 (volume OR capacity)) OR AB ((tidal OR lung*) N2 (volume OR capacity))  OR TI (burn* N2 (inhalation OR pulmonary OR lung*)) OR AB (burn* N2 (inhalation OR pulmonary OR lung*))  OR TI "Sudden Death" OR AB "Sudden Death"  OR TI "work of breathing" OR AB "work of breathing"  OR TI "breathing work*" OR AB "breathing work*" |
| **Warfare**  (44,288) | MH "War" OR MH "Biological Warfare" OR MH "Chemical Warfare" OR MH "Chemical Warfare Agents" OR MH "Terrorism+"  OR TI ((Biological OR chemical OR "Mass Destruction") N2 (Weapon* OR warfare)) OR AB ((Biological OR chemical OR "Mass Destruction") N2 (Weapon* OR warfare))  OR TI ((Warfare OR Biothreat OR Biological) N2 Agent*) OR AB ((Warfare OR Biothreat OR Biological) N2 Agent*)  OR TI ((Asymmetric OR conventional OR "Non-conventional") N2 war*) OR AB ((Asymmetric OR conventional OR "Non-conventional") N2 war*)  OR TI ("terrorist* attack*" OR Terrorism OR bioterror* OR bio-terror* OR peace-keeping OR peacekeeping OR military)  OR AB "terrorist* attack*" OR Terrorism OR bioterror* OR bio-terror* OR peace-keeping OR peacekeeping OR military)  OR TI "political assassination*" OR AB "political assassination*"  OR TI "intelligence operation*" OR AB "intelligence operation*"  OR TI "mass casualt*" OR AB "mass casualt*" |
| **Chemical Threat**  (44,788) | MH Herbicides  OR MH "Biohazard Release" OR MH "Chemical Hazard Release"  OR MH "Hydrocarbons, Halogenated"  OR MH "Ammonia" OR MH "Chlorine" OR MH "Hydrogen Sulfide" OR MH "Hydrogen Cyanide"  OR MH "Dioxins"  OR MH "Cyanides"  OR MH "Phosphorus Compounds" OR MH "Phosphorus"  OR MH "Formaldehyde"  OR MH "Toxins+"  OR TI (Nerve N2 (Agent* OR gas)) OR AB (Nerve N2 (Agent* OR gas))  OR TI ((blister OR choking OR blood OR neurotoxic OR suffocating) N2 agent*) OR AB ((blister OR choking OR blood OR neurotoxic OR suffocating) N2 agent*)  OR TI sarin OR AB sarin  OR TI soman OR AB soman  OR TI "agent VX" OR AB "agent VX"  OR TI "mustard gas" OR AB "mustard gas"  OR TI "S-mustard" OR AB "S-mustard" OR TI "n-mustard" OR AB "N-mustard"  OR TI lewisite OR AB lewisite  OR TI ammonia OR AB ammonia  OR TI Vesicant* OR AB Vesicant*  OR TI Napalm OR AB Napalm  OR TI "Agent Orange" OR AB "Agent Orange"  OR TI "Hydrogen Sulfide" OR AB "Hydrogen Sulfide"  OR TI Methylamine OR AB Methylamine  OR TI "Sulfur Dioxide" OR AB "Sulfur Dioxide"  OR TI Phosgene OR AB Phosgene  OR TI phosphine OR AB phosphine  OR TI "Carbon Tetrachloride" OR AB "Carbon Tetrachloride"  OR TI Chloride* OR AB Chloride*  OR TI "sulfur dioxide" OR AB "sulfur dioxide"  OR TI Dioxin* OR AB Dioxin*  OR TI Cyanide* OR AB Cyanide*  OR TI Cyclohexanes OR AB Cyclohexanes  OR TI Chloroacetophenone OR AB Chloroacetophenone  OR TI Chlorobenzylidenemalonitrile OR AB Chlorobenzylidenemalonitrile  OR TI "tichloromethyl chloroformate" OR AB "tichloromethyl chloroformate"  OR TI Phosphorus OR AB Phosphorus  OR TI Thermine OR AB Thermine  OR TI Formaldehyde OR AB Formaldehyde  OR TI Herbicide* OR AB Herbicide*  OR TI Oxime* OR AB Oxime*  OR TI hemotoxic OR AB hemotoxic  OR TI "biological toxin*" OR AB "biological toxin*"  OR TI ricin OR AB ricin  OR TI botuli* OR AB botuli*  OR TI "clostri-perfringens toxin" OR AB "clostri-perfringens toxin"  OR TI mycotoxin* OR AB mycotoxin*  OR TI palytoxin* OR AB palytoxin*  OR TI saxitoxin* OR AB saxitoxin*  OR TI "staphylococcus enterotoxin*" OR AB "staphylococcus enterotoxin*"  OR TI tetrodoxin* OR AB tetrodoxin*  OR TI "SEB intoxicat*" OR AB "SEB intoxicat*" |
| **Cochrane - 27** | |
| **Respiratory**  (126,600) | [mh "Respiration Disorders"]  OR [mh "Respiratory Mucosa"]  OR [mh "Respiratory Physiological Phenomena"]  OR [mh "Respiratory Muscles"]  OR [mh "Bronchioles"]  OR [mh "Signs and Symptoms, Respiratory"]  OR [mh "Airway Management"]  OR [mh "Vital Signs"]  OR [mh "Thoracic Wall"]  OR [mh Death, Sudden]  OR [mh "Lung Volume Measurements"]  OR [mh "Burns, Inhalation"]  OR [mh "work of breathing"]  OR Respirat*:ti,ab,kw OR Pulmonary:ti,ab,kw  OR "mucociliary clearance":ti,ab,kw  OR "lung compliance":ti,ab,kw  OR (Airway NEAR/2 (Remodeling OR Resistance OR Management)):ti,ab,kw  OR "diaphragm paralysis":ti,ab,kw  OR bronchiole*:ti,ab,kw  OR "Vital Sign*":ti,ab,kw  OR (Wall NEAR/2 (Thoracic OR Chest)):ti,ab,kw  OR "sudden death":ti,ab,kw  OR (burn* NEAR/2 (inhalation OR pulmonary OR lung*)):ti,ab,kw  OR ((tidal OR lung*) NEAR/2 (volume OR capacity*)):ti,ab,kw  OR "work of breathing":ti,ab,kw OR "breathing work*":ti,ab,kw |
| **Warfare**  (2764) | [mh "Chemical Warfare"]  OR [mh "Biological warfare"]  OR [mh "Biological Warfare Agents"]  OR [mh "Chemical Warfare Agents"]  OR [mh ^"Weapons of Mass Destruction"]  OR ((Biological OR chemical OR "mass destruction") NEAR/2 (Weapon* OR warfare)):ti,ab,kw  OR ((Warfare OR Biothreat) NEAR/2 Agent*):ti,ab,kw  OR ("terrorist* attack*" OR Terrorism OR bioterror* OR bio-terror* OR peace-keeping OR peacekeeping OR military):ti,ab,kw  OR ((Asymmetric OR conventional OR Non-conventional) NEAR/2 war*):ti,ab,kw  OR "political assassination*":ti,ab,kw  OR "intelligence operation*":ti,ab,kw  OR "mass casualt*":ti,ab,kw |
| **Chemical Threat**  (38,386) | [mh "Nerve Agents"]  OR [mh "Agent Orange"]  OR [mh "Chemical Hazard Release"]  OR [mh "Biohazard Release"]  OR [mh "Sarin"]  OR [mh "Soman"]  OR [mh "Mustard Gas"]  OR [mh "Hydrogen Cyanide"]  OR [mh ^"Hydrocarbons, Halogenated"]  OR [mh Ammonia]  OR [mh "Chlorides"]  OR [mh methylamine]  OR [mh "Hydrogen Sulfide"]  OR [mh "Sulfur Dioxide"]  OR [mh "Phosgene"]  OR [mh phosphine]  OR [mh "Carbon Tetrachloride Poisoning"]  OR [mh "Carbon Tetrachloride"]  OR [mh "Dioxins"]  OR [mh "Dioxins and Dioxin-like Compounds"]  OR [mh Cyclohexanes]  OR [mh "omega-Chloroacetophenone"]  OR [mh "o-Chlorobenzylidenemalonitrile"]  OR [mh Phosphorus]  OR [mh "Phosphorus Compounds"]  OR [mh Oximes]  OR [mh Toxins, Biological]  OR [mh "Herbicides"]  OR [mh "Formaldehyde"]  OR [mh "Ricin"]  OR (Nerve near/2 (Agent* OR gas)):ti,ab,kw  OR "hydrogen sulfide":ti,ab,kw  OR ("Agent Orange" OR Vesicant* OR Napalm OR "Chemical Hazard Release" OR "biohazard release" OR ammonia OR chloride* OR methylamine OR "sulfur dioxide" OR Phosgene OR phosphine OR "carbon tetrachloride" OR dioxin* OR cyanide* OR cyclohexane* OR Chloroacetophenone OR Chlorobenzylidenemalonitrile OR phosphorus OR Thermine OR Formaldehyde OR Oxime* OR herbicide* OR tabun OR sarin OR soman OR "toxic gas" OR "mustard gas" OR "s-mustard" OR "n-mustard" OR lewisite OR "trichloromethyl chloroformate" OR diphosgene OR chloropilkrin OR ricin OR "clostri-perfringens toxin*" OR mycotoxin* OR palytoxin* OR saxitoxin* OR "staphylococcus enterotoxin*" OR tetrodoxin* OR botuli* OR "halogenated hydrocarbon*" OR hemotoxic):ti,ab,kw  OR ((blood OR blister OR choking OR neurotoxic OR suffocating OR VX) near/2 agent*):ti,ab,kw  OR "SEB intoxicat*":ti,ab,kw |

**Table S1 -Query used in the 5 databases**

NOTE The numbers appearing next to the database name corresponds to the combination of the three concepts as of November 6, 2018. The number of results for each concept is shown in parentheses.
